# Pharmacological pitfalls in inpatient lung transplant care and management of medication risks

**DOI:** 10.3389/fphar.2026.1794658

**Published:** 2026-05-29

**Authors:** Rolf Dieterich, René Hage, Macé M. Schuurmans, Jérôme Bonzon

**Affiliations:** 1 Department of Clinical Pharmacology and Toxicology, University Hospital Zurich, Zurich, Switzerland; 2 Faculty of Medicine, University of Zurich, Zurich, Switzerland; 3 Department of Pulmonology, University Hospital Zurich, Zurich, Switzerland

**Keywords:** clinical pharmacology, drug–drug interactions, immunosuppression, lung transplantation, tacrolimus

## Abstract

Pharmacotherapy after lung transplantation is complex and prone to drug–drug interactions, dosing errors, and toxicity. We retrospectively analysed 259 comments from weekly interdisciplinary rounds at the University Hospital Zurich between September 2022 and April 2024 to identify common pharmacological pitfalls in inpatient lung transplant recipients. The cohort included 62 patients (mean age 56.1 years; 40.3% female). Most comments concerned potential risks rather than confirmed harm: 54.4% were classified as potential side effects and 21.6% as potential loss of effectiveness. Interactions were the most frequent subcategory (45.6%), followed by dosage problems (14.3%). Tacrolimus was the most commonly commented on drug, often due to interactions with metamizole/dipyrone, resulting in fluctuating tacrolimus levels. Other frequent issues included QTc-prolonging combinations (e.g., domperidone with escitalopram or amiodarone in addition to itraconazole) and reduced levothyroxine absorption due to divalent cations. Only five comments reflected actual adverse outcomes, indicating overall high drug safety. These findings are consistent with the literature describing the importance of systematic pharmacological review and interdisciplinary collaboration to improve medication safety and standardise care in lung transplant recipients.

## Introduction

Lung transplantation is an established treatment for selected patients with end-stage lung disease. This is relevant for organ procurement, careful recipient selection, and optimal postoperative treatment (Schuurmans et al., 2013). There have been ongoing changes in the selection of recipients and donors, the surgical procedures, and the pharmacological treatment. Particularly, immunosuppression is of great importance for long-term outcomes (Hage et al., 2025; Ivulich et al., 2018). Compared to 20 years ago, survival after lung transplantation has improved, increasing from 4.7 years to 6.7 years over the past decade. However, this still varies depending on the indication for transplantation. For patients who survive the first year post-transplant, the current median survival ranges from 8.9 (Chambers et al., 2019) to 11.1 years (Bos et al., 2020). Among other factors, this depends on the underlying disease and comorbidities, which are again relevant for appropriate recipient selection (Kotloff and Thabut, 2011; Adegunsoye et al., 2017; Featherstone, 2011).

Pharmacological management after lung transplantation is particularly challenging. Lifelong immunosuppression with drugs such as calcineurin inhibitors must be carefully balanced against risks of rejection, infection, toxicity, and long-term complications (Featherstone, 2011). Infections and graft failure pose the greatest risk for death within the first year after transplantation (Perch et al., 2022). Calcineurin inhibitors also pose a heightened risk for drug–drug interactions due to their extensive metabolism (Adegunsoye et al., 2017). In addition, Ehrsam et al. (2022) found that the most frequent comorbidities associated with poorer survival were congestive heart failure, osteoporosis, gastro-oesophageal reflux, systemic hypertension, and mild diabetes. Specifically, comorbidities that have already led to systemic or end-stage damage may worsen the outcome but can also constitute a contraindication. Their treatment should precede transplantation (Weill et al., 2015). Thus, lung transplant recipients frequently receive multiple co-medications, leading to substantial polypharmacy. Therefore, a clinical pharmacologist forms an important part of the interdisciplinary post-transplant treatment team (McDermott and Girgis, 2018; Thompson et al., 2013).

Among other clinical or digital rounds, there is a weekly interdisciplinary round for all inpatient lung transplant recipients during the initial post-operative hospitalisation at the University Hospital Zurich, which is held in person with the projection of clinical parameters, images, and drugs. This round usually includes thoracic surgeons, pulmonologists, critical care physicians, infectious disease specialists, and clinical pharmacologists. All prescriptions are routinely checked by the team of clinical pharmacologists, especially for potential prescribing errors (interactions, dosage, indications/contraindications, and Galenics). These comments are collected in an internal database during every round. Some such examples include the following:

- Interactions mostly refer to drug–drug interactions
- Dosage refers to over- or under-dosing
- Indication/contraindication refers to deviations from the approved drug label
- Galenics refers to improper handling of pharmaceutical formulations, such as the splitting of tablets that are not intended to be split

While medication safety in solid-organ transplantation has been addressed in kidney and liver transplant populations (Shawaqfeh et al., 2023; Bril et al., 2016), there is a limited systematic analysis of the pharmacological pitfalls specifically in inpatient lung transplant recipients. Most studies in lung transplantation focus on potential drug–drug interactions or individual drug classes and are often conducted outside structured interdisciplinary rounds (Duwez et al., 2020; Xie et al., 2018). Therefore, the primary aim of this study was to systematically identify and characterise the frequent pharmacological pitfalls in the initial postoperative course of inpatient lung transplant recipients, as documented during interdisciplinary clinical rounds. By analysing prospectively recorded pharmacological comments, we sought to describe common risk patterns related to drug interactions, dosing, contraindications, and monitoring and thereby provide a descriptive basis for targeted standardisation and quality improvement in post-transplant pharmacotherapy.

Our dataset provides real-world evidence from a prospectively logged pharmacology review in inpatients conducted during inpatient rounds. In contrast to algorithmic potential drug–drug interaction screening alone, we classify the comments into actionable categories (interaction, dosing, contraindication, and monitoring) and map them to high-risk immunosuppressive agents (e.g., tacrolimus with -azoles or metamizole), thereby operationalising medication safety principles specifically for lung transplant recipients. This positions our work alongside, but distinct from, existing drug–drug interaction summaries and pharmacist-impact studies by quantifying pitfalls observed at the point of care during rounds and translating them into a standardised postoperative workflow.

## Patients and methods

All comments from the interdisciplinary rounds between 13 September 2022 and 23 April 2024 were recorded in an internal Microsoft Access® database used for quality control by the Department of Clinical Pharmacology and Toxicology. Data collection began with the introduction of the weekly interdisciplinary round, including clinical pharmacology, on the above-mentioned date. The interdisciplinary pharmacology rounds included lung transplant recipients during their initial post-surgical hospitalisation.

The number of comments per round and the number of patients discussed varied due to the different transplant activities during the study period. Some patients were readmitted to the hospital for different reasons during the study period. Since medication issues vary depending on the time-since transplantation, these patients were not retained in the analysis.

**FIGURE 1:**
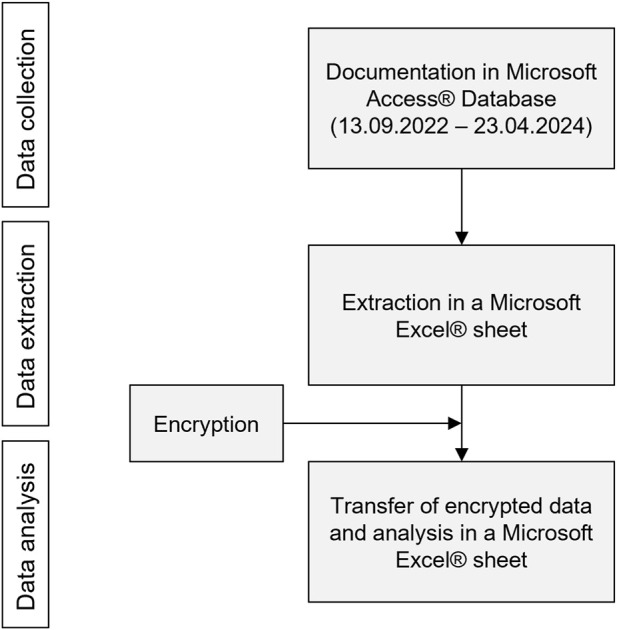
Process of data collection, extraction, and analysis.

To analyse this dataset, the principal investigator (last author) of this study performed data extraction from the Microsoft Access® database into a Microsoft Excel® spreadsheet. The extracted dataset included all comments, their categories, the date of the round, sex, and year of birth. Each comment was assigned a unique identification number. A separate key document enabled secure pseudonymisation of the data. The re-identification key was created and maintained exclusively by the project lead (last author) and stored in a password-protected folder on the institution’s internal server, accessible only through his personal credentials. The analysis itself was conducted by the first author. The flow of data collection, extraction, and analysis is shown in Figure 1.

The comments are collected in a structured way to facilitate evaluation. Although there are internal guidelines regarding the structure of the comments, some individual variation remains. Each entry in the Microsoft Access® database contains the name, date of birth, individual case number, and date of the round. Subsequently, the entry is assigned to one of the categories listed in Table 1.

**TABLE 1:** Categories and subcategories of pitfalls.

Category	Subcategory
General information	Dosage problem
Potential side effect	Interaction
Side effect	Galenics
Potential loss of effectiveness	Therapeutic drug monitoring
Loss of effectiveness	Indication
Costs	Contraindication
Hepatotoxic side effects	Mistake
​	Safety monitoring

The expectation of a side effect or loss of effectiveness is considered a potential event. Only measurable laboratory, clinical, or diagnostic findings were classified as side effects or loss of effectiveness. The classification into categories is subject to the pharmacologist’s medical assessment and is based on internal guidelines; however, for historical reasons, no strict cut-off points have been defined.

The comments are further specified by choosing one or more of the subcategories listed in Table 1.

Due to the historical database design, only two medications can be selected in a drop-down list and are labelled “medication” and “co-medication.” These terms reflect a documentation convention rather than the pharmacological hierarchy or clinical relevance. The designation “medication” refers to the drug that represents the primary focus of the pharmacological assessment in a given comment, whereas “co-medication” denotes a second drug considered relevant to the identified issue (e.g., through a drug–drug interaction or additive risk). Importantly, a given drug may appear as either “medication” or “co-medication” in different comments, depending on the clinical context and focus of the assessment.

For the quantitative analyses, only medications recorded in these structured fields were included. A free-text field allows the exact description of the pitfall. It refers to the selected drug combination in the drop-down lists and provides additional details beyond these structured fields. Because some comments concern diagnostic suggestions rather than prescribing errors, completion of categories, subcategories, or medications is not mandatory.

We chose to analyse only the (co-)medications selected in the drop-down lists in a systematic way as they are considered the relevant medications involved in the respective comment. The free-text documentation differed in wording. We decided to standardise the free text entries and group them accordingly to provide further detailed information. Care was taken not to alter the technical content. As the technical content was already aligned with the internal guidelines, verification was not performed; however, the standardisation process was supervised by the principal investigator.

The explorative retrospective analysis was conducted using the descriptive statistical functions available in Microsoft Excel®. For all results containing decimal numbers, values were rounded to one decimal place where appropriate.

The primary endpoint was defined as the medication most frequently commented on, including the corresponding comment category. The secondary endpoints included basic demographic characteristics of the cohort, such as mean age, sex category distribution, and potential sex-specific differences in pharmacotherapy.

Due to the nature of the database, which records only comments but not patients without interventions, patient-level stratification was not possible.

## Results

**FIGURE 2:**
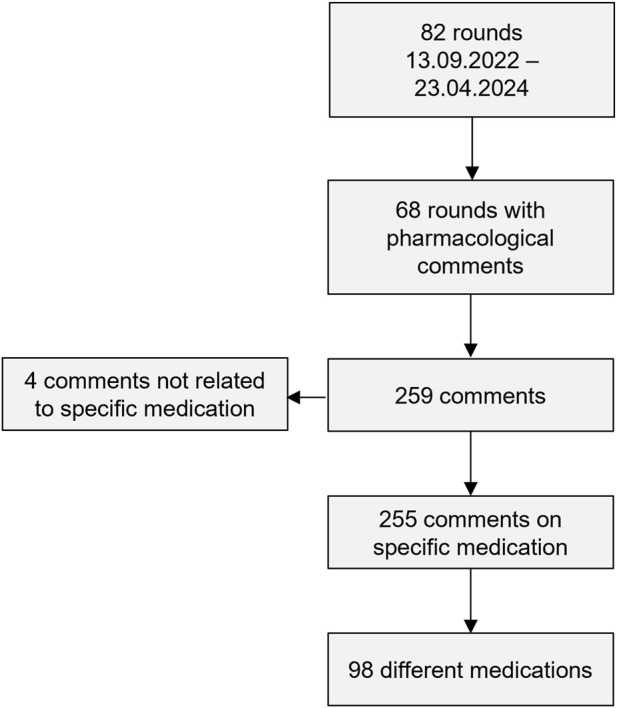
Composition of comments.

A total of 259 comments were extracted from the database, of which 255 comments referred to a specific medication, as shown in Figure 2. Consequently, the analyses of drug interactions and pitfalls were based on these 255 comments. The comments had been created and documented between 13 September 2022 and 23 April 2024. These comments were documented during 82 interdisciplinary rounds. In 68 rounds, the pharmacological pitfalls were detected and commented on. An average of 3.6 patients were assessed per round. Of all patients discussed in each round, an average of two patients required specific pharmacological comments.

The study population included 62 patients, comprising 40.3% female and 59.7% male patients. The mean age was 56.1 years ±10.1 years (range 22–73 years; Table 2).

**TABLE 2:** Baseline characteristics of the population percentages refer to the total number of commented patients (n = 62).

Characteristic	​	n (%)
Total number of commented patients	​	62 (100)
Age* *At the time of the first round	18–34 years 35–49 years 50–64 years ≥65 years Mean years ± SD	2 (3.2) 11 (17.7) 36 (58.1) 13 (21) 56.1 ± 10.1
Sex	Male Female	37 (59.7) 25 (40.3)
Average patient cases per round	​	3.6 (100)
Average patients commented on (clinical pharmacology) per round	​	2 (55.5)

All comments were allocated to a single category. Our analysis revealed that most comments were categorised as “potential side effect.” These comments referred to prescriptions or drug combinations that could potentially cause adverse effects. This was followed by “potential loss of effectiveness” and “general information.” Only three comments were categorised as “side effect,” and two were categorised as “loss of effectiveness/”. The categories are listed in Table 3.

**TABLE 3:** Categories of pitfall percentages refer to the total comments (n = 259).

Category	n (%)
Total number of comments	259 (100)
Potential side effect Potential loss of effectiveness General information Hepatotoxic side effect Side effect Loss of effectiveness Cost	141 (54.4) 57 (21.6) 49 (18.9) 7 (2.7) 3 (1.2) 2 (0.8) 0

Table 4 presents the results of the analysis of the subcategories. Combinations of the subcategories could also be assigned. The most common subcategory was “interaction” with (45.6%), followed by dosage problems, safety monitoring, and contraindications. Together, these account for more than three-quarters of all the identified issues.

**TABLE 4:** Subcategories of pitfall and possible combinations percentage calculated from the total comments (n = 259).

Subcategory	n (%)
Total number of comments	259 (100)
Interaction Dosage problem Safety monitoring Contraindication Galenics Indication Indication + safety Mistake Dosage problem + contraindication Interaction + contraindication Therapeutic drug monitoring Mistake + interaction Dosage problem + safety	118 (45.6) 37 (14.3) 32 (12.4) 30 (11.6) 15 (5.8) 13 (5) 4 (1.5) 3 (1.2) 2 (0.8) 2 (0.8) 1 (0.4) 1 (0.4) 1 (0.4)

**FIGURE 3:**
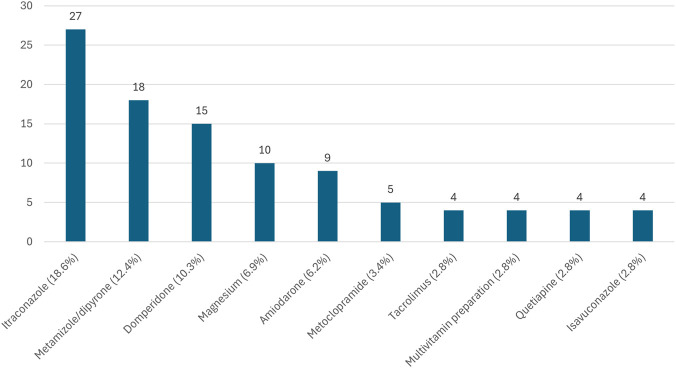
Medication with the most comments (n = 127 out of 255 medication mentions). Percentages in the figure refer to n = 255.

**FIGURE 4:**
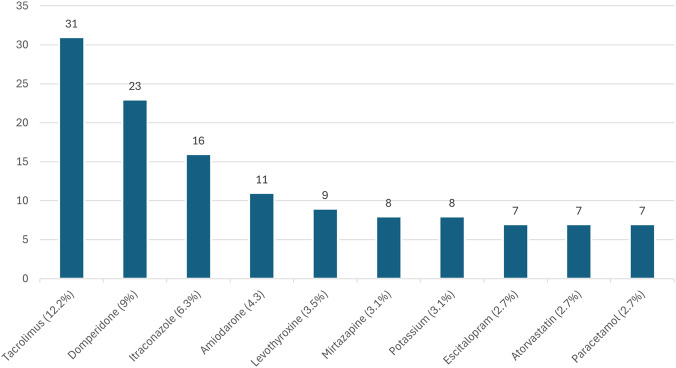
Co-medication with the most comments (n = 100 out of 145 co-medication mentions). Percentages in the figure refer to n = 145.

A total of 98 distinct medications were selected as either primary therapy (“medication”) or adjunctive therapy (“co-medication”), as shown in Figures 3, 4. Tacrolimus was the most frequently mentioned drug, followed by domperidone and itraconazole. Itraconazole was the most frequently mentioned co-medication, followed by metamizole/dipyrone and domperidone. Overall, itraconazole was the most commented-on drug, followed by domperidone and tacrolimus. In 145 comments, two drugs were recorded, whereas 110 comments involved only one medication.

The most common combination of drugs was tacrolimus with metamizole/dipyrone, followed by tacrolimus with amiodarone, domperidone with itraconazole, and amiodarone with domperidone. The other frequent combinations are listed in Table 5.

**TABLE 5:** Fifteen most commonly commented combinations of medication and co-medication. Percentages refer to the number of documented medication–co-medication combinations (n = 145). Not every drug listed in Figures 3, 4 appears here because this table is restricted to the most frequent combinations.

Medication	Co-medication	n (%)
Total number of combinations		145 (100)
Tacrolimus Levothyroxine Domperidone Itraconazole Amiodarone Mirtazapine Escitalopram Atorvastatin	Metamizole/dipyrone Amiodarone Divalent cations Itraconazole Metoclopramide Atorvastatin Tamsulosin Quetiapine Pantoprazole Magnesium Domperidone Itraconazole Itraconazole Domperidone Itraconazole	16 (11) 5 (4.1) 9 (6.2) 5 (3.4) 4 (2.8) 3 (2.1) 2 (1.4) 2 (1.4) 2 (1.4) 2 (1.4) 5 (3.4) 3 (2.1) 3 (3.1) 6 (4.1) 4 (2.8)

The most detailed insight into the pitfalls was obtained by analysing the free-text comments together with their category and subcategory, as shown in Table 6. The most common comment concerned potential loss of effectiveness of tacrolimus due to interaction with metamizole/dipyrone prescribed on demand (PRN), leading to fluctuating tacrolimus levels. The second most common comment concerned potential loss of effectiveness of levothyroxine due to its interaction with divalent cations. The potential side effect of the interaction between escitalopram and domperidone represented the third most frequent comment, followed by the potential side effects of the interactions between amiodarone and domperidone and tacrolimus and amiodarone. Discrepancies between Tables 5, 6 arise due to several reasons. First, one comment on tacrolimus and metamizole/dipyrone referred to a different pharmacological issue than the other comments. Second, due to the historical structure of the database, medication was documented as a regularly prescribed and regularly dosed compound influenced by a co-medication. For the free text analysis, these were analysed together. Finally, all preparations containing divalent cations were grouped together, as they represent the same interaction mechanism with levothyroxine and the same clinical pitfall.

**TABLE 6:** Most common comments in free text. Counts shown represent raw frequencies within the free-text dataset (total structured free-text entries = 259). Percentages are not shown because the denominators vary.

Medication combination and category with the subcategory of pitfall	Comment	n
Tacrolimus + metamizole/dipyrone Potential loss of effectiveness + interaction	PRN or short-term regular prescription of metamizole/dipyrone can lead to considerable changes in tacrolimus-levels due to the induction of CYP3A4. We, therefore, recommend either a long-term regular prescription or to discontinue metamizole/dipyrone	15
Levothyroxine ± calcium/magnesium/multivitamin preparation/food Potential loss of effectiveness + interaction	Due to possible complex formation with divalent cations and for a better resorption, levothyroxine should be taken 30 min before all other medication and with a time gap of 4 h to divalent cations	9
Escitalopram + domperidone Potential side effect + interaction	Regular ECG monitoring is recommended due to the increased risk of QTc interval prolongation	8
Amiodarone + domperidone Potential side effect + interaction	Regular ECG monitoring is recommended due to the increased risk of QTc interval prolongation	6
Tacrolimus + amiodarone Potential side effect + interaction	This combination can lead to increased levels of tacrolimus, which is probably due to the inhibition of P-gp/ABCB1 and CYP3A4 through amiodarone	5
Paracetamol Hepatotoxic side effect + contraindication and dosage problem	Paracetamol usage should be stopped or limited in its dosage with increasing liver enzymes and/or chronic alcohol abuse	5
Domperidone + itraconazole Potential side effect + contraindication	The combination of domperidone and strong inhibitors of CYP3A4 (itraconazole in this case) is formally contraindicated	4
Domperidone + metoclopramide Potential side effect + interaction	Combination of domperidone and metoclopramide is not recommended due to a similar mechanism of action	4

## Discussion

In this retrospective analysis of prospectively documented pharmacological pitfalls, we describe the frequency and types of pitfalls identified during interdisciplinary rounds. The most common comments concerned potential side effects from drug–drug interactions between tacrolimus and metamizole/dipyrone.

We provide systematic and prospectively documented comments on pharmacological pitfalls from weekly interdisciplinary rounds in an inpatient lung-transplant cohort, representing an under-reported yet clinically relevant data source. Thereby, this study adds several aspects to the current literature by offering a rarely reported but clinically relevant source of data. By outlining a categorisation of comments, our analysis may inform future outcome-linked evaluations (e.g., mapping comments to clinical outcomes) and could facilitate the development of a repeatable workflow applicable to an intensive care unit (ICU) and ward rounds.

The baseline characteristics of our population are comparable to those reported in the annual report of the Organ Procurement and Transplantation Network and Scientific Registry of Transplant Recipients (Valapour et al., 2023). The registry reports 63.8% male and 36.2% female patients receiving a lung transplant in 2021 compared to 59.7% male and 40.3% female patients in our cohort. The age distribution differs to a varying extent: 18–34 years, 5.5% (registry) vs. 3.2% (our cohort); 35–49 years, 11.8% vs. 17.7%; 50–64 years, 46% vs. 58.1%; and ≥65 years, 36.7% vs. 21%. Our data represent inpatients (and a few outpatient follow-up visits), rather than all annual transplant recipients of a certain year. This may explain the lower proportion of recipients aged ≥65 years in our cohort relative to the cited registry (Valapour et al., 2023); however, differences in study design and inclusion periods limit direct comparability. The mean age of 56.1 years compares to 57 years in a population from the University of California (Gaboyan et al., 2024).

In 68 of 82 interdisciplinary rounds, an average of 3.6 patients were discussed weekly. Pharmacological pitfalls were identified on average in two patients. Thus, pharmacological pitfalls were detected only in 55.5% of inpatient lung transplant recipients. This quantifies the frequency of the comments in our rounds. No external benchmark was available for comparison.

Most comments were categorised as potential side effects or potential loss of effectiveness, predominantly due to interactions. Nearly half of them fell into the interaction subcategory, followed by dosage issues (often related to renal function). Only three comments were classified as side effects, and two were classified as loss of effectiveness. No comment was categorised as cost, which is typically used in other populations when standard medications were replaced by more expensive alternatives. The two comments on loss of effectiveness referred to tacrolimus levels below the target despite dose adjustments. The three comments regarding side effects concerned toxic tacrolimus levels in two cases and a toxic cefepime level in one case. Therefore, most comments reflected potential risks, mainly interactions or dosage. Confirmed adverse events were rare.

The most commented-on medication was tacrolimus, reflecting its central role in postoperative immunosuppression. Tacrolimus, a calcineurin inhibitor, is part of standard triple immunosuppression with mycophenolate mofetil and glucocorticoids (Hage et al., 2025; McDermott and Girgis, 2018). Its broad drug–drug interaction and side-effect profile have to be considered (Hage et al., 2024). The second most commented medication was domperidone. The QTc-prolonging potential is high and, therefore, a commonly commented-on medication. It is part of the standard post-transplant treatment (Hage et al., 2025). The third most commented-on medication was itraconazole, which is used for antifungal prophylaxis. In our setting, itraconazole is administered for prolonged and uninterrupted periods. Co-administration of itraconazole is associated with higher tacrolimus exposure, which in clinical practice often results in lower tacrolimus doses (due to the inhibition of CYP3A4 and P-glycoprotein (P-gp)). However, this also results in a broad interaction spectrum (Hage et al., 2024). As a common co-medication, metamizole/dipyrone is one of the most commented-on drugs. Metamizole/dipyrone has the potential to decrease tacrolimus levels through the induction of CYP3A4. When prescribed, PRN metamizole/dipyrone has been observed to be associated with fluctuating tacrolimus levels (Sigaroudi et al., 2019). This finding partly overlaps with results from other studies analysing drug–drug interactions after transplantations (Duwez et al., 2020; Sigaroudi et al., 2019; Nelson et al., 2022; Katz, 1999). Our dataset did not include clinical outcomes of the detected pitfalls.

Due to the global differences in drug approval, one cannot compare all medications to each other in different countries. Domperidone, an antiemetic drug, which acts as a peripheral dopamine-antagonist, is not approved in the United States of America. The use of metamizole/dipyrone also varies considerably worldwide.

In our transplant centre, ciclosporin was used as the first-line calcineurin inhibitor for 3 decades but has recently been replaced by tacrolimus. Ciclosporin is mainly used when tacrolimus-based immunosuppression leads to complications such as posterior reversible encephalopathy, chronic allograft dysfunction, or recurrent acute allograft dysfunction. It is used less frequently when therapeutic tacrolimus levels cannot be achieved or when contraindications or toxicity occurs. Tacrolimus has largely replaced ciclosporin as the primary calcineurin inhibitor in many centres, reflecting the reports of improved rejection-free and overall survival (Dellgren et al., 2024) and of its more favourable interaction profile (Thompson et al., 2013).

Tacrolimus combined with metamizole/dipyrone was the most common drug combination, although individual drugs were more frequently commented on. This is because some combinations are not routinely flagged as pitfalls within our standard operating procedure (SOP). The combinations associated with the highest number of comments were tacrolimus and itraconazole (interacting pharmacokinetically, as described above) and domperidone and tacrolimus (interacting pharmacodynamically, with the potential to increase the QTc interval). The combination with the second-highest number of comments involved interactions between levothyroxine and divalent cations present in calcium supplements, multivitamins, or certain foods, which can reduce the levothyroxine levels. Although this finding is not related to a specific post-transplantation medication, it illustrates a recurrent issue observed in clinical practice regarding the importance of the appropriate treatment of pre-existing comorbidities.

One of our secondary endpoints was to evaluate sex-specific differences in pharmacotherapy. Sex-stratified tables can be found in the supplementary material. Concerning the categories, there was no considerable difference. In both groups, interactions were by far the most common subcategory. However, in male patients, the second most common subcategory was safety monitoring, followed by dosage problems. In female patients, the second most common subcategories were dosage problems and contraindications. In addition, the distribution of the most common medication and co-medication in the sex-specific context differed. In male patients, medications most frequently commented on were tacrolimus, followed by itraconazole, domperidone, and amiodarone. In female patients, the most frequently commented-on medications were tacrolimus, domperidone, levothyroxine, and escitalopram. Notably, in women, half of the most frequently commented medications are related to the treatment of comorbidities. The sex-stratified distributions align with known epidemiology (e.g., atrial fibrillation with use of amiodarone in men and hypothyroidism and the use of antidepressants in women). Our data are descriptive and were not designed to test sex-related differences in outcomes (BFS BfS, 2024; Chaker et al., 2022; Hindricks et al., 2021).

Immunosuppressive therapy is challenging with regard to drug–drug interactions, particularly considering the available and commonly used drugs. This challenge increases when integrating it into the pharmacological regimen of multi-morbid patients receiving underlying medications (McDermott and Girgis, 2018).

The cost-effectiveness and added value of pharmacological assessments in this population are being discussed. Duwez et al. (2020) analysed the role of clinical pharmacists in outpatient lung transplant recipients. Our findings are consistent with their report that the involvement of pharmacologists, similarly to clinical pharmacists, identifies drug-related problems in transplant recipients. In our dataset, we described the comment frequencies; however, we did not measure the downstream clinical effects. Interestingly, they identified dosage and indication as the most common pitfalls, whereas interactions followed by the dosage were the most frequent in our dataset. Similar to our study, they found that immunosuppression was the largest area of medication errors. Embedding a pharmacology expert in structured interdisciplinary rounds is consistent with the literature describing associations between the involvement of medication experts and fewer prescribing errors. Our round-based model is, therefore, consistent with best-practice guidance and guideline expectations for transplant teams (Duwez et al., 2020).

Regarding the limitations of this study, the design limits its information value, as it is a single-centre retrospective data analysis. In order to determine the specific effect of an intervention, such as the pharmacological assessment of medication, a prospective randomised approach would be required.

Due to the nature of our Microsoft Access® database, the underlying data offers only limited possibilities for a precise evaluation of our comments. As a result, the free-text fields, which may have contained potentially more detailed information, were not systematically analysed but were grouped together to provide more detailed insight into the corresponding interaction pairs. In addition, it was not possible to cluster the comments by specific patients or to analyse the key results at the patient level. These findings characterise the observed pattern of comments in our setting and do not establish causal relationships with clinical outcomes. This limits the overall interpretability of our findings.

Additionally, this study might not offer generalisable results because potentially sicker patients with more comorbidities are being evaluated more often. Therefore, we provide more data on this population to allow comparisons. The data stem from a population where only a very small proportion of the transplant recipients have cystic fibrosis as an underlying condition.

Finally, it should be noted that the data source consists exclusively of interdisciplinary round comments documented by the Department of Clinical Pharmacology and Toxicology. As a result, the dataset is subject to individual variation, although internal guidelines for these rounds exist. This may introduce selection bias and over- and underreporting.

In summary, our analysis describes the most frequently observed pharmacological pitfalls in inpatient lung transplant recipients discussed during interdisciplinary rounds, predominantly involving tacrolimus interactions and dose-adjustment issues. Most comments reflected the potential risk rather than documented harm. These observations may inform local SOPs and educational priorities; however, causal inferences or clinical outcomes cannot be drawn from this descriptive study design. Future work should link categories to standardised harm indices and patient-level outcomes.

Weekly interdisciplinary rounds for all inpatient lung transplant recipients provide a structured opportunity to review pharmacological pitfalls and potential drug interactions at the University Hospital Zurich. In addition to other SOPs for lung transplant recipients, a brief internal workflow has been implemented for immediate postoperative treatment, addressing the most important aspects of pharmacological management. This workflow primarily addresses our ICU as all lung transplant recipients remain in the specialised transplant ICU during the postoperative period. It also covers issues arising in the specialised post-transplant surgical ward prior to discharge to rehabilitation or home. This workflow standardises pharmacological decision-making within the team. Its impact on the safety or outcomes was not assessed in this analysis.

## Data Availability

The raw data supporting the conclusions of this article will be made available by the authors upon reasonable request, without undue reservation.

## References

[B1] Adegunsoye A. Strek M. E. Garrity E. Guzy R. Bag R. (2017). Comprehensive care of the lung transplant patient. Chest 152 (1), 150–164. 10.1016/j.chest.2016.10.001 27729262 PMC6026268

[B2] BFS BfS (2024). Gesundheit taschenstatistik 2024. Neuchâtel: Bundesamt für Statistik (BFS).

[B3] Bos S. Vos R. Van Raemdonck D. E. Verleden G. M. (2020). Survival in adult lung transplantation: where are we in 2020? Curr. Opin. Organ Transpl. 25 (3), 268–273. 10.1097/MOT.0000000000000753 32332197

[B4] Bril F. Castro V. Centurion I. G. Espinosa J. Keller G. A. Gonzalez C. D. (2016). A systematic approach to assess the burden of drug interactions in adult kidney transplant patients. Curr. Drug Saf. 11 (2), 156–163. 10.2174/157488631102160429003742 27194037

[B5] Chaker L. Razvi S. Bensenor I. M. Azizi F. Pearce E. N. Peeters R. P. (2022). Hypothyroidism. Nat. Rev. Dis. Prim. 8 (1), 30. 10.1038/s41572-022-00357-7 35589725

[B6] Chambers D. C. Cherikh W. S. Harhay M. O. Hayes D. Jr. Hsich E. Khush K. K. (2019). The international thoracic organ transplant registry of the international society for heart and lung transplantation: thirty-Sixth adult lung and heart-lung transplantation Report-2019; focus theme: donor and recipient size match. J. Heart Lung Transpl. 38 (10), 1042–1055. 10.1016/j.healun.2019.08.001 31548030 PMC6816340

[B7] Dellgren G. Lund T. K. Raivio P. Leuckfeld I. Svahn J. Holmberg E. C. (2024). Effect of once-per-day tacrolimus *versus* twice-per-day ciclosporin on 3-year incidence of chronic lung allograft dysfunction after lung transplantation in scandinavia (ScanCLAD): a multicentre randomised controlled trial. Lancet Respir. Med. 12 (1), 34–44. 10.1016/S2213-2600(23)00293-X 37703908

[B8] Duwez M. Chanoine S. Lepelley M. Vo T. H. Pluchart H. Mazet R. (2020). Clinical evaluation of pharmacists' interventions on multidisciplinary lung transplant outpatients' management: results of a 7-year observational study. BMJ Open 10 (11), e041563. 10.1136/bmjopen-2020-041563 33247028 PMC7703423

[B9] Ehrsam J. P. Schuurmans M. M. Laager M. Opitz I. Inci I. (2022). Recipient comorbidities for prediction of primary graft dysfunction, chronic allograft dysfunction and survival after lung transplantation. Transpl. Int. 35, 10451. 10.3389/ti.2022.10451 35845547 PMC9276940

[B10] Featherstone B. (2011). A rough guide to transplant medicines. Pharm. J. 287 (7678), 539. 10.1211/PJ.2021.1.66816

[B11] Gaboyan S. Afshar K. Chin J. Verheyden J. Sullivan L. Marley S. (2024). “Demographics of lung transplant recipients participating in explant tissue collection at the university of California San Diego,” in B74 breathing new life: lung transplant pathogenesis AND mechanisms. American Thoracic Society, A4403–A.

[B12] Hage R. Schuurmans M. M. Roeder M. Damm D. (2025). Lung transplant manual, 1 ed2022, 103.

[B13] Hage R. Bonzon J. Schuurmans M. M. (2024). Direct oral anticoagulation in lung transplant recipients. Exp. Clin. Transplant. 22 (4), 249–257. 10.6002/ect.2023.0338 38742314

[B14] Hindricks G. Potpara T. Dagres N. Arbelo E. Bax J. J. Blomström-Lundqvist C. (2021). 2020 ESC guidelines for the diagnosis and management of atrial fibrillation developed in collaboration with the european association for cardio-thoracic surgery (EACTS): the task force for the diagnosis and management of atrial fibrillation of the european society of cardiology (ESC) developed with the special contribution of the european heart rhythm association (EHRA) of the ESC. Eur. Heart J. 42 (5), 373–498. 10.1093/eurheartj/ehaa612 32860505

[B15] Ivulich S. Westall G. Dooley M. Snell G. (2018). The evolution of lung transplant immunosuppression. Drugs 78 (10), 965–982. 10.1007/s40265-018-0930-6 29915895

[B16] Katz H. I. (1999). Drug interactions of the newer oral antifungal agents. Br. J. Dermatol. 141, 26–32. 10.1046/j.1365-2133.1999.00011.x 10730911

[B17] Kotloff R. M. Thabut G. (2011). Lung transplantation. Am. Journal Respiratory Critical Care Medicine 184 (2), 159–171. 10.1164/rccm.201101-0134CI 21471083

[B18] McDermott J. K. Girgis R. E. (2018). Individualizing immunosuppression in lung transplantation. Glob. Cardiol. Sci. Pract. 2018 (1), 5. 10.21542/gcsp.2018.5 29644232 PMC5857067

[B19] Nelson J. Kincaide E. Schulte J. Hall R. Levine D. J. (2022). Immunosuppression in lung transplantation. Handb. Exp. Pharmacol. 272, 139–164. 10.1007/164_2021_548 34796380

[B20] Perch M. Hayes D. Jr. Cherikh W. S. Zuckermann A. Harhay M. O. Hsich E. (2022). The international thoracic organ transplant registry of the international society for heart and lung transplantation: thirty-ninth adult lung transplantation report-2022; focus on lung transplant recipients with chronic obstructive pulmonary disease. J. Heart Lung Transpl. 41 (10), 1335–1347. 10.1016/j.healun.2022.08.007 36050206 PMC10257980

[B21] Schuurmans M. Benden C. Inci I. (2013). Practical approach to early postoperative management of lung transplant recipients. Swiss Med. Wkly. 143. 10.4414/smw.2013.13773 23572438

[B22] Shawaqfeh M. S. Alangari D. Aldamegh G. Almotairi J. Bin Orayer L. Albekairy N. A. (2023). Unveiling medication errors in liver transplant patients towards enhancing the imperative patient safety. Saudi Pharm. J. 31 (11), 101789. 10.1016/j.jsps.2023.101789 37799574 PMC10550402

[B23] Sigaroudi A. Jetter A. Mueller T. F. Kullak-Ublick G. Weiler S. (2019). Severe reduction in tacrolimus concentrations with concomitant metamizole (dipyrone) therapy in transplant patients. Eur. J. Clin. Pharmacol. 75 (6), 869–872. 10.1007/s00228-019-02635-y 30694339

[B24] Thompson M. L. Flynn J. D. Clifford T. M. (2013). Pharmacotherapy of lung transplantation: an overview. J. Pharm. Pract. 26 (1), 5–13. 10.1177/0897190012466048 23204148

[B25] Valapour M. Lehr C. J. Schladt D. P. Smith J. M. Goff R. Mupfudze T. G. (2023). OPTN/SRTR 2021 annual data report: lung. Am. J. Transplant. 23 (2), S379–S442. 10.1016/j.ajt.2023.02.009 37132345 PMC9970343

[B26] Weill D. Benden C. Corris P. A. Dark J. H. Davis R. D. Keshavjee S. (2015). A consensus document for the selection of lung transplant candidates: 2014—an update from the pulmonary transplantation council of the international society for heart and lung Transplantation. J. Heart Lung Transplant. 34 (1), 1–15. 10.1016/j.healun.2014.06.014 25085497

[B27] Xie Y. Dilibero D. Chang D. H. (2018). Review of major drug-drug interactions in thoracic transplantation. Curr. Transplant. Rep. 5, 220–230. 10.1007/s40472-018-0200-2

